# Antibodies, Nanobodies, or Aptamers—Which Is Best for Deciphering the Proteomes of Non-Model Species?

**DOI:** 10.3390/ijms21072485

**Published:** 2020-04-03

**Authors:** Poshmaal Dhar, Rasika M. Samarasinghe, Sarah Shigdar

**Affiliations:** 1 School of Medicine, Deakin University, 75 Pigdons Road, Waurn Ponds, Victoria 3216, Australia; posh.dhar@deakin.edu.au (P.D.); r.samarasinghe@deakin.edu.au (R.M.S.); 2 Centre for Molecular and Medical Research, Deakin University, 75 Pigdons Road, Waurn Ponds, Victoria 3216, Australia

**Keywords:** antibodies, aptamers, nanobodies, proteins, proteome, reagents

## Abstract

This planet is home to countless species, some more well-known than the others. While we have developed many techniques to be able to interrogate some of the “omics”, proteomics is becoming recognized as a very important part of the puzzle, given how important the protein is as a functional part of the cell. Within human health, the proteome is fairly well-established, with numerous reagents being available to decipher cellular pathways. Recent research advancements have assisted in characterizing the proteomes of some model (non-human) species, however, in many other species, we are only just touching the surface. This review considers three main reagent classes—antibodies, aptamers, and nanobodies—as a means of continuing to investigate the proteomes of non-model species without the complications of understanding the full protein signature of a species. Considerations of ease of production, potential applications, and the necessity for producing a new reagent depending on homology are presented.

## 1. Introduction

**Figure 1:**
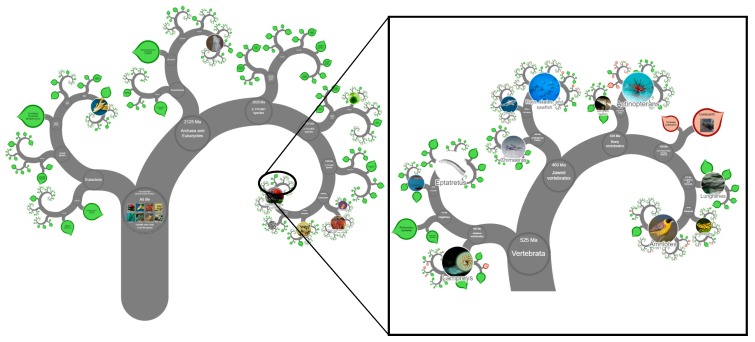
Current representation of the Tree of Life from Open Tree of Life (opentreeoflife.org). Of the more than two million known species on this planet, only ≈70,000 are vertebrates. Only a limited number of these have genomes sequenced and fewer have information regarding their proteomes. A high resolution image of this figure can be found at http://www.onezoom.org/life.html/@biota=93302#x1033,y1463,w1.9286.

In the era of personalized medicine and the ability to have your own sample sent for genetic testing, there is still a perceived lack of progression in treating human disease. While genetic mutations are valuable for determining the presence (or absence) of a number of diseases, it is the proteins that are transcribed from the genes that are responsible for the incidence of diseases, as these proteins become the functional part of the cell. While the human genome project was instigated in 1990, technological advancements have meant that analyzing the protein signature, or proteome, has lagged behind. Since then, researchers have managed to achieve a commendable feat by sequencing the genomes of around 15,000 species. However, unfortunately, the number of complete proteomes is far shorter. With the number of eukaryotic species on this planet being in the tens of millions and the diversity of prokaryotic species reaching the trillions, we are still a long way from truly understanding the species-richness and variety of biology (Figure 1). We are slowly grasping the importance of understanding these functional units of different species and how these can aid in our understanding of disease and pathological processes [1]. While most of the research has been conducted on mammalian species, we have gained a good deal of knowledge in development and disease from other vertebrate species, with certain species of fish being utilized more and more over the last few years [2].

## 2. The Zebrafish as a Model Organism for Human Disease

Though the zebrafish has only recently gained popularity as a model organism, we have already made vital discoveries using zebrafish to understand developmental pathways, disease progression, and therapeutic strategies for diseases and disorders [3]. While there are a plethora of reasons why zebrafish serve as an ideal model for investigating immune cell development and disease progression, such as their transparent embryonic and larval developmental stages (reviewed in [4]), the primary reason for their widespread use is that their genome has been sequenced, making it easier to make significant comparisons between the genome and the proteome. This makes it easier to decipher results of studies undertaken in zebrafish to human development and disease processes, significantly improving our understanding of human illnesses.

There have been numerous studies that have investigated the proteome of zebrafish [5,6,7,8]. Most of these studies have utilized mass spectrometry and liquid chromatography-tandem mass spectrophotometry approach. In situ hybridization using RNA probes, which were developed nearly 10 years ago [9], have been extensively used in this field to study developmental pathways and immunological pathways in health and disease. While these approaches have expanded our understanding of the role of immune-modulatory and regulatory proteins in Zebrafish, the field has been impeded due to a lack of reliable antibodies or probes to investigate the pathways involved in health and disease. There have been attempts to generate reliable antibodies and probes to overcome this limitation. One such progress has been in the field of neurobiology, wherein researchers have demonstrated a panel of antibodies that are effective in visualizing neural receptors in zebrafish [10]. A monoclonal antibody against a tumor suppressor protein has also been generated [11]; however, the commercial use is yet to be elucidated. Thus, despite the availability of a complete genomic sequence, one of the major limitations has been the lack of appropriate antibodies or probes to study disease progression in zebrafish models.

What of other piscine species and what we can learn from them? Fish are the most numerous and phylogenetically diverse group of vertebrates, encompassing over 20,000 species [12,13]. They are also the oldest extant vertebrates, having inhabited the earth for more than 500 million years [14,15,16,17]. Given this great diversity, along with a diverse pattern of ageing, a small size, and ease of cultivation, there is much that can be gathered from these species. This will open new avenues for us to understand the different biological processes, including disease modelling, response to external stimuli (such as infections, environmental changes), ageing, and response to drugs [18,19,20].

The molecular pathways involved in immunity are highly conserved, with the innate immune system dating back to the early metazoans, and the acquired immune system dating back to the origin of the jawed vertebrates about 450 million years ago [21,22]. When a pathogen invades a host, it stimulates a number of these conserved pathways [22,23], which leads to the altered expression of key genes that serve as sensitive markers of infection. The innate immune response is generally mounted early in response to invading organisms and includes a complex network of molecules and cells, both specific and non-specific, which operate to clear the pathogen from its host. Some of the main cells involved in these innate responses are leucocytes (including neutrophils, macrophages, and dendritic cells). Their primary role is to phagocytose and digest the pathogen to present it to adaptive immune cells (such as T and B cells), or kill/eliminate the pathogen by producing antimicrobial molecules and immune-modulatory proteins. Some of the molecules include antibacterial peptides (cathelicidins, human β defensins), lysozyme, transferrin, complement, acute-phase proteins, prostaglandins (PGE2), reactive oxygen intermediates (ROI), cyclooxygenase-2 (COX-2), cytokines, chemokines, and lectins [24,25]. While a vast amount of information regarding the immune system in mammals exists, transferring this knowledge to fish has led to some difficulties. This is due to a number of issues, not least of which is the fact that fish species live in their own unique environment. However, the newly acquired ability in molecular biology to sequence and clone genes has become a powerful system for the study of vertebrate immune development and disease [25]. Indeed, even though fish immunology at the molecular level is becoming well understood, functional biology of fish immune cells, both *in vivo* and *in vitro*, is still in its infancy [25]. Undoubtedly, this field has witnessed significant improvements over the last ten years, but there is still much that we do not know and the majority of research to date has focused on commercially important species and biosecurity [26].

Comparative immunologists have been interested in the immune system of fish and how it has similarities and differences with higher vertebrates [27,28,29]. However, the lagging step has been the production of suitable reagents to recognize piscine targets [30]. While the genetics and transcripts for a number of piscine species have been described, this does not provide sufficient functional knowledge of pathways activated during disease processes. To do that, knowledge of a particular protein sequence is required in order to generate reagents. With the availability of CRISPR-based technology, analyzing immunological pathways has become possible in the zebrafish,. Overall, while there is a large scope for generation of tools to study protein–protein interactions in fish species, the very slow process, even with technological advancements, to add knowledge regarding both piscine and broader species, and expanding the field of comparative immunology remains a key challenge.

## 3. Proteomes of Larger Species

The first step to understanding the complexities of the proteome across multiple animals, including humans, is to sequence the genomes. The Earth BioGenomes project was announced in 2018 to “sequence life for future life” [31]. The initial goal of this project was to generate annotated chromosome-scale reference assemblies for a minimum of one reference species across each of the approximately 9000 eukaryotic taxonomic families. Once this is completed, it will be possible to look for percentage homology amongst species and begin the process of assessing which species may have similar proteomes. This information, probably more so than with the use of smaller vertebrates or non-mammalian models, will provide more knowledge on predicting which animal model systems are more appropriate for studying specific disease states and lead to the development of a more refined list of species specific models rather than the reliance on mice and rats.

As discussed earlier, the molecular pathways involved in immunity are highly conserved across species, indicating that responses that are generated by hosts (human or non-human species) to infections are extremely likely to be similar. However, when attempting to model the response to an infection, or even how an infection spreads, the choice of animal model is complex. It is important to note that during an immune response to an infection or allergen, there is not only involvement of the immune system, but also the interplay with other pathways, including the neuroendocrine and physiological systems. The differences (and similarities) in these other physiological systems between humans and other species are bound to have an effect on the overall understanding of a particular disease or treatment. For new and emerging infections, an animal model which can predict the pathogenesis of the disease and respond in a similar manner to therapeutics or vaccines is required [32]. An example would be the use of ferrets for modelling influenza infections. Ferrets are one of the few animal models that exhibit most of the clinical symptoms observed in humans, and they can be easily infected with human influenza viruses and transmit the disease efficiently between each other [33]. While the number of studies using ferrets as an animal model for influenza has increased since 2008, it was not until 2011 that the proteome was deposited in UniProt.org. The ferret is also now being used as a model for cystic fibrosis research [34]. While there is a paucity of reagents [33], the National Ferret Resource and Research Center has compiled a list of commercially available antibodies that are applicable to ferret proteins.

Staying on the theme of animal models for human disease, it is not only knowledge of proteomes that can aid us in understanding which model to choose, but also which model not to use in pre-clinical studies. For example, mice and pigs have been used in cardiac research but with limited success. A recent study sought to elucidate the proteomes of cardiac development in the mouse, pig, and *Xenopus* to better understand the most appropriate model for modelling cardiac disease. Proteins known to be expressed in humans were also found to be enriched in frogs, but surprisingly not in mice or pigs [35]. It is unsurprising then that frogs may find their way into more translatable research in the future [36], especially given that half of human genes differ from their mouse orthologs in different developmental trajectories, including more than 200 disease genes associated with brain, heart, and liver diseases [37]. These differences can impact the proteomes, and therefore phenotypes, between humans and mice. This leads to the necessity of reliable reagents needed for interrogating the protein signatures of animal models for human disease.

When it comes to assessment of other animals, specifically in regard to the animals that are important to farming, proteome research has been very limited (Table 1). The majority of the research has largely focused on animal products rather than the animal itself. This can be attributed to two main reasons: high costs and a lack of genomic data. Where there has been a focus on animals, it has been driven by predictors of food quality or the detection of infectious diseases. In instances where traditional proteome research techniques, such as shotgun-based approaches, have been utilized in this field, these have missed proteins of relevance to research and health, such as cytokines and their receptors. This may be due to the low concentration of these immune-modulatory proteins in the biological samples when compared to other proteins, which increases the chances of them being undetected during analysis. When considering food security for the future, it is important that a different approach is considered to identify disease biomarkers and biomarkers relevant to health and immune function [38,39].

It is worth noting that while there is limited information available for animals important to humans as food sources, there is a lack of credible information available for native species. The Peptide Atlas has information on cows, horses, mice, pigs, rats, and zebrafish. UniProt contains more data for native species, though it mostly has not been reviewed. From an Australian perspective, proteomes are available for the platypus and the Tasmanian devil, but not the echidna, koala, kangaroo, or possum, leading to an extreme paucity of data for species of ecological significance.

## 4. Towards a Better System of Detecting Proteome Signatures

So if we are no closer to understanding the proteomes of all the species on this planet, or even those that are classed as important to humans, such as farm animals, pets, and wildlife, are there ways we can adapt current technology to interrogate proteins that appear to be similar or related to diseases across species? The main stay in human disease research over the past 40 years has been monoclonal antibodies [52]. These have proven to be very effective in diagnostic applications where the antibody has been fully validated and quality controls are in place to ensure each batch of antibody performs to the strict requirements [53]. However, in research applications there have been several limitations, notwithstanding the reproducibility crisis that has been placed at the feet of reagents, and antibodies in particular, in a high percentage of cases [54].

**Figure 2:**
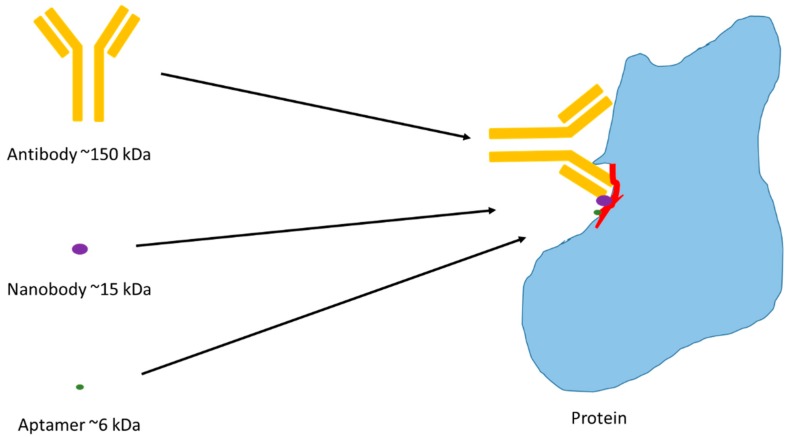
Binding of antibody, nanobody, and aptamer to a protein, highlighting the size differences between the three potential binding reagents. In proteins where the homologous region (highlighted in red) between species is small, only part of the antibody may fit, leading to poor species cross-reactivity. The nanobody and aptamer are 10 and 20 times smaller, respectively, and are more likely to cross-react between species.

In order to generate an antibody, purified protein is required and this protein needs to be immunogenic in order to generate an immune response once the protein is injected into an animal host [30]. Following the time required to initiate an immune response and generate antibodies, the B-cells of the spleen are removed and fused with myeloma cells to form hybridomas which are then tested to identify the ones which produce the best monoclonal antibodies for their target [55]. This process has limitations, as you need to know what your target, or protein, is to begin with. Now, with the advent of in vitro combinatorial display libraries, it may be possible to identify antibody fragments that bind to a particular protein. However, the peptide fragments may not necessarily show reactivity to other species, as the peptides are selected on the Fc region of typically human IgG [56]. There are commercial phage display libraries derived from mouse, rabbit, chicken, camel, and llama (creative-biolabs.com), and there may be some cross-reactivity to other species, though the ability of these combinatorial libraries to be used for native species is limited given the probable differences in genome and proteome homology (Figure 2). Additionally, if an antibody is discovered from one of these libraries, its applications may be limited to diagnostic applications, rather than theranostic, due to their likely immunogeneic nature.

The discovery that camels have IgG-like material in their serum that showed similarity to antibodies but were devoid of light chains suggested new opportunities for the engineering of antibodies [57]. These camelid single chain antibodies show a high sequence homology to the variable domains of heavy chains in humans (80 %) [58]. The highly stable single antigen binding domain is much smaller than a conventional antibody, a key benefit, as they are only about 15 kDa, which has led to them being known as nanobodies [59,60]. This may allow them to bind to antigenic sites not recognized by antibodies or to “hidden” antigenic sites [58]. Due to their similarity to human immunoglobulins, they have a low immunogenic profile in humans [61], although the same probably could not be said for other species, which presents a limitation for wider therapeutic applications. In terms of selecting nanobody phage libraries, the process is similar to the phage display libraries used to select for antibodies. Interestingly, these nanobody libraries have recently been used to select for cell surface receptors in situ as well as intracellular targets [62,63], which does open up possibilities for selecting nanobodies against unknown targets on particular sub-populations of cells. Additionally, nanobodies have been generated that are cross-reactive to human and murine proteins [64] or all *Trypanosoma* species [65].

Given the challenges associated with the use of antibodies and nanobodies in therapeutic applications, and lack of cross-species reactivity, other classes of molecules are required to advance our knowledge in other species. A comparatively recent advancement in the field has been the generation of aptamers, which are single stranded DNA or RNA sequences that bind to their targets in a similar manner to antibodies and nanobodies—through shape recognition to their target binding sites. In much the same way as combinatorial libraries of antibodies and nanobodies are used, combinatorial libraries of nucleic acid sequences are used to select aptamers that bind to a target. This process, known as the systematic evolution of ligands by exponential enrichment (SELEX), was first described in 1990 [66,67,68] and involves a library of approximately 10^14^ individual sequences that are incubated with a target over iterative rounds to “evolve” a number of sequences that bind with high specificity and sensitivity. These binding sequences are typically 6–12 kDa in size and therefore have similar properties to nanobodies in that they can bind to antigenic sites that are not recognized by antibodies (Figure 2). For pathways that are highly conserved [69], it is possible that a “binding site” might be large enough and homologous enough that antibodies may show cross-reactivity amongst species. The smaller the binding molecule though, the more likely the recognition. However, where there is divergence and homology is decreased, the binding site may only be amenable to very small binders that have been generated to other species’ proteins. In a similar manner to nanobodies being smaller versions of antibodies, an aptamer is fairly easy to truncate to its smallest functional form, which may increase its possibility of binding to multiple species [70,71,72]

Of note for the use of combinatorial libraries, it is possible to take the sequence of the targeting moiety and mutate this sequence with multiple mutations to find one that will bind to a different species’ protein, though depending on the format of the assay and the knowledge of the other protein, this may not be possible [73]. However, there are several other protocols available that might be amenable for developing targeting agents. For example, a protocol amendment for aptamer development that reacts to human and porcine thrombin was first described in 2001 [74]. This process was coined “toggle SELEX” and involved interchanging the protein in selection rounds between the two. There have since been several adaptations to this protocol to generate cross-species binding aptamers [75,76,77].

## 5. Fishing for Homology: Potential Protocols for Developing Targeting Agents

It is still possible to determine cross-species affinity without complete knowledge of the genomes and proteomes. If sufficient protein can be purified, this can be the starting point for screening against commercially available antibodies. A study by Villarreal and colleagues purified protein from zebrafish to clone into the recombinant SUMO solubility tag expression system to test an antibody from Abcam that had been generated for a human protein [78]. Cross-species reactivity of antibodies has also been shown between fish species [79]. Studies using aptamers and nanobodies are limited in the literature, though this is an ever-expanding space. However, most of the research has been limited to human and murine cross-reactivity for translational studies, with very few studies looking at other species. Interestingly, and highlighting the point earlier made about the size of the binding region, there have been a few studies that have truncated aptamers to enhance their cross-species reactivity. For example, Dhiman et al. truncated an aptamer from 40 nucleotides to 26 nucleotides to recognize toxins from two different snake species [70]. One study has investigated the ability of three different aptamers to bind to thrombin in six different species (human, bovine, porcine, rabbit, rat, and mouse). The shortest aptamer sequence, comprising 15 nucleotides, had the least variation in activity compared to the other two longer sequences [80]. These studies, taken together, suggest that truncating aptamers to their smallest possible functional units would enhance researchers’ abilities to interrogate cellular pathways in non-model animal models.

**Figure 3:**
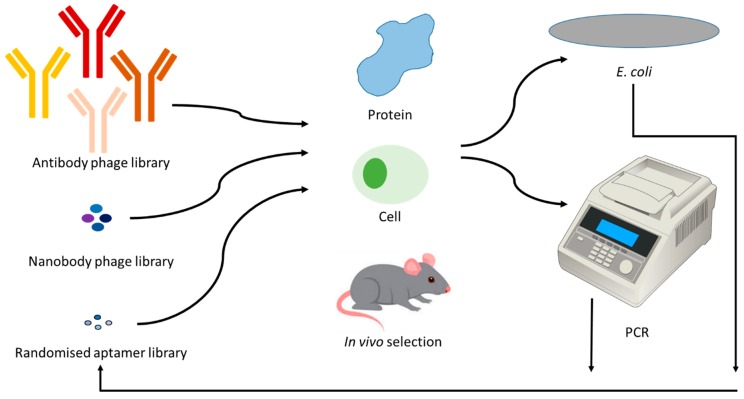
Diagrammatic representation of the process involved in the generation of specific binding reagents. Combinatorial phage libraries of antibodies or nanobodies, or combinatorial randomized sequences of aptamers are incubated with a target, followed by removal of unbound species via washing. The bound sequences are propagated in *Escherichia coli* or via PCR. Re-incubation of these enriched species is continued for 3–7 iterative rounds.

Purified protein or a known protein sequence is not necessarily a limiting factor for the generation of a targeting ligand. Selection can be attempted without knowing the precise target if sufficient materials, such as specific cells, are available. For example, nanobody libraries have been used to select for cell surface receptors [63], though in this case the proteins were expressed in cells prior to selection. This can also be performed using aptamer libraries [81] though it is also possible to “fish” for targets on whole cells or organisms [82,83]. It is even possible to develop antibodies against targets on whole cells using the combinatorial libraries [84]. A schematic is provided in Figure 3 to demonstrate the selection process. The protocol used for generating nanobodies to cell surface receptors used a process similar to SELEX whereby the nanobody library was incubated with target cells for iterative rounds and bound species were re-amplified using *Escherichia coli* rather than using PCR to re-amplify the bound nucleic acid species [63,85]. This process can be expanded on through the use of tissue sections, and both antibodies and aptamers have been generated to rare cells using both frozen [86,87] and paraffin embedded tissue as the antigen substrate [88]. Where homology is suggested, rather than undergoing a complete selection of a full combinatorial library, it is possible to use a directed library. For example, mutations can be introduced into the known binding sequence to generate a much smaller library that may require only one incubation step to detect a targeting ligand. This is an adaptation of the process underpinning panning for better affinity or novel therapeutic mechanisms [89,90]. This process may be simpler with aptamers, due to the smaller sequences and nucleic acid nature which allows base pair switching or error-prone PCR to introduce mutations [73].

Where no knowledge of the homology of the protein, or even the protein target, is available, it may still be possible to develop targeting regents. Traditionally, antibodies and nanobodies have been generated following an immunization event, with antibodies/nanobodies being collected from the B-cells of the spleen. An improvement on this has been the injection of combinatorial libraries into target animals to collect those that have bound to a target organ or cell type [91], a method that has also been achieved with aptamer libraries [92]. This method has the benefit of providing aptamers or antibodies that are relevant for in vivo applications, though it does present some technical challenges.

## 6. In Vitro Applications: Which is Best?

Given that antibody, nanobody, and aptamer selection can all now be based on combinatorial libraries, are there benefits to using one over the other? This comes down to what the future application(s) may be (Table 2). Antibodies and nanobodies are proteins, and so have requirements for limited changes to pH, salt composition, and temperature, as extreme conditions can denature them. As well, selection conditions need to be close to physiological conditions due to the risk of affecting the protein structure. This means that the possibility of developing antibodies or nanobodies to targets in complex media or non-physiological conditions is limited. Aptamers are not limited by these parameters, and selection conditions can be modified to ensure the aptamers will work in any application [93]. Indeed, with the use of modified nucleotides, it is possible to select aptamers in very acidic or alkaline conditions [94,95]. One other major drawback of antibodies and nanobodies over aptamers is the cost involved in generating them [96].

While traditional antibody tests, such as enzyme linked immunosorbent assays (ELISAs) and immunophenotyping by flow cytometry are completed in a laboratory environment, there is a requirement for point of care testing (POCT) and rapid diagnostic tests that span all species [97,98,99]. Going back to the protein nature of antibodies and nanobodies, these require special conditions to be used in the field for POCT. For example, transport needs to be refrigerated and antibodies/nanobodies need to be kept cool to ensure they remain functional, whereas aptamers are stable at higher temperatures [53,100]. Additionally, while aptamers have been used to replace antibodies in typical laboratory tests, they have also come into their own in a number of in vitro diagnostic assays that would be easily adapted to in field or POCT [101,102,103,104].

An avenue where aptamers are being utilized due to the diverse advantages they offer is the rapidly emerging field of handheld electrochemical sensors. Aptamers are smaller in size, have a much more flexible nature, and have better conductivity than antibodies [97,105]. Add in the issues of stability and protein biofouling, and it not strange that aptamers have gained popularity in this field in recent years [106]. Indeed, aptamer based electrochemical sensors have been used in many fields, such as clinical diagnostics, food analysis, and environmental science [107]. While these devices have been used to detect small molecules, proteins, and even cells in clinically relevant environments, they have not yet been used in non-model organisms [97,105,107]. As researchers start to investigate these non-model organisms to understand development or disease pathology, and to understand the health and well-being of species, the field of aptamer research is likely to change and expand.

## 7. Conclusions

This review has been kept deliberately broad due to the nature of this area of research being almost all encompassing. With so many species on the planet still waiting for complete genomes, let alone proteomes, to be completed so we have a better idea of the Tree of Life, there are some reagents that can be directly used to interrogate the proteins of other species. However, for the vast majority of species, new reagents need to be generated to advance knowledge. These reagents can be antibodies, nanobodies, or aptamers, with the choice being ease of development, ease of use, and future applications. Some may be more likely to cross-react across species than others, depending on the size of target binding site. However, with antibodies dominating the diagnostic and research field for 40 years, it may now be necessary to investigate the benefits offered by nanobodies and aptamers as alternative affinity reagents to decipher similarities and differences in evolutionary pathways and the implications of these developmental changes between species.

## Figures and Tables

**Table 1:** Studies that have compared species’ proteomes.

Tested Tissue/Cells/ Protein	Species	Technique	Total No of Similar Proteins between Species	Reference
Kidney cortical transporters	Human, monkey, dog, rat, and mouse	LC–MS/MS	19	[40]
Liver and kidney efflux drug transporters	Human, monkey, rat, dog	Isotope dilution nano LC-MS/MS	4 specific transporters: MDR1/P-gp, BCRP, MRP2 and MRP3	[41]
Liver microsomes	Human, rat, mouse	2D-(SCX-RP)-LC–MS/MS	704	[42]
Milk casein micelles	Holstein cows, buffaloes, Jersey cows, yaks, goats, camels, and horses	LC-MS/MS	25	[43]
Milk fat globule membrane	Human, cow, goat and yak	LC–MS/MS	50	[44]
Milk fat globule membrane	Humans, Holstein and Jersey cows, buffaloes, yaks, goats, camels, horses	LC–MS/MS	399	[45]
Pancreatic beta cells	Human and rat	label-free LC-MS/MS	185	[46]
Pancreatic cells	Mouse, rat and human	SDS-PAGE (gel) coupled with LC-MS/MS		[47]
Platelets	Human, Rat	SDS-PAGE–LC MS/MS	837	[48]
Saliva	Human, Dog, Glires, Sheep, cattle, horses	LC–MALDI, SDS-PAGE–LC MS/MS	13	[49]
Seminal plasma	Alpaca, cattle, horse, sheep, pig, goat and camel	SDS-PAGE LC–MS/MS and 2D–LC–MS/MS	302	[50]
Sperm	Rodents and ungulates	LC-MS/MS	623	[51]

BCRP: breast cancer resistance protein; LC-MS/MS: liquid chromatography with tandem mass spectrometry; MALDI: matrix-assisted laser desorption/ionization; MDR: multidrug resistance protein; MRP: multidrug resistance-associated protein; P-gp: P-glycoprotein; SCX-RP: strong cation exchange reverse phase; SDS-PAGE: sodium dodecyl sulfate–polyacrylamide gel electrophoresis.

**Table 2:** Pros and cons of using antibodies, nanobodies, or aptamers in research.

Conditions	Antibodies	Nanobodies	Aptamers
Use in physiological conditions (pH, temp, etc)	✓	✓	✓
Use in non-physiological conditions	X	X	✓
Complex target selection	✓	✓	✓
Stability in wide temperature range	X	X	✓
Immunogenicity	✓	✓	X/limited

## Abbreviations

COX-2	Cyclooxygenase
MYA	Million years ago
PGE2	prostaglandins
POCT	Point of care test
ROI	Reactive oxygen species
SELEX	Systematic evolution of ligands by exponential enrichment
